# Editorial: Aging and male hypogonadism

**DOI:** 10.3389/fendo.2023.1207907

**Published:** 2023-05-19

**Authors:** Kai Xia, Jiajie Yu, Guihua Liu, Haolin Chen, Renshan Ge, Chunhua Deng

**Affiliations:** ^1^ Center for Stem Cells and Regenerative Medicine, Zhongshan School of Medicine, Sun Yat-sen University, Guangzhou, China; ^2^ Department of Urology and Andrology, The First Affiliated Hospital of Sun Yat-sen University, Guangzhou, China; ^3^ Reproductive Medicine Center, Sixth Affiliated Hospital of Sun Yat-sen University, Guangzhou, China; ^4^ Department of Gynecology and Obstetrics, The Second Affiliated Hospital and Yuying Children’s Hospital of Wenzhou Medical University, Wenzhou, China; ^5^ Department of Anesthesiology, Perioperative Medicine, Zhejiang Province Key Lab of Anesthesiology, The Second Affiliated Hospital and Yuying Children’s Hospital of Wenzhou Medical University, Wenzhou, China

**Keywords:** hypogonadism - hypotestosteronemia - androgen deficiency, aging, male, Leydig cell, testosterone

Male hypogonadism, characterized by testosterone deficiency, is common in males over 40 years old (1, 2). Male hypogonadism can lead to sexual and reproductive dysfunction, as well as age-related diseases such as cardiovascular diseases and metabolic disorders, seriously affecting male quality of life and health. Currently, exogenous testosterone replacement therapy is the main clinical treatment for male hypogonadism, but it can only partially alleviate symptoms and cannot cure the disease. Besides, it also has obvious side effects, such as increasing the risk of cardiovascular diseases, exacerbating obstructive sleep apnea syndrome, and increasing the risk of prostate cancer (3, 4). Therefore, it is necessary to explore safer and more effective methods of testosterone supplementation. Leydig cells (LCs) located in the interstitium of the testis and are the main source of testosterone. Their dysfunction is the core mechanism of age-related male hypogonadism (5, 6). Exploring the intrinsic mechanism of Leydig cell aging can help us better understand male hypogonadism and develop new treatment strategies.

Age-related male hypogonadism is the result of both primary and secondary factors, however, the association of chemical exposure remains unclear. Phthalates are widely used plasticizers and can be found in many products, including food packaging, toys and medical devices (7). A recent retrospective study, utilizing data from the National Health and Nutrition Examination Survey (NHANES), found a positive association between exposure to Di(2-ethylhexyl) phthalate (DEHP) metabolites and the risk of age-related male hypogonadism in men over 40 years old (Liu et al.). The odds ratio increased with the concentration of DEHP metabolites, and a dose-dependent effect was observed. Liu et al. highlight for the first time the role of phthalates exposure in the etiology of age-related male hypogonadism, contributing to our understanding of this disease.

In addition to the complex pathophysiology, the diagnostic criteria and symptom assessment of age-related male hypogonadism have been controversial. Insulin-like peptide 3 (INSL3) is a hormone secreted by mature LCs, and is commonly used to assess the functional status of LCs (8). Ivell et al. collected and analyzed data from over 3000 men in the European Male Aging Study (EMAS) cohort, finding that INSL3 is a more effective marker than testosterone (T) for evaluating primary hypogonadism, as well as secondary and compensated hypogonadism. Elevated INSL3 levels are associated with age-related diseases such as cardiovascular disease and hypertension. However, this study also suggests that INSL3 may play a causal role in promoting healthy bone metabolism. These findings suggest that INSL3 may be a useful biomarker for identifying individuals at risk of developing age-related male hypogonadism and related comorbidities.

Testosterone deficiency associated with aging can lead to a decline in semen quality, indicating impaired spermatogenesis. Conventional semen quality assessments primarily include quantifiable parameters such as total sperm count, sperm volume, and progressive sperm motility, but fail to provide insight into the underlying biological significance of these parameters. Recently, metabolomics and proteomics analyses were conducted on semen from aging males (Guo et al.). Guo et al. established a biomarker panel consisting of four metabolites (Pipamperone, 2,2-Bis(hydroxymethyl)-2,2’,2’’-nitrilotriethanol, Arg-Pro, Triethyl phosphate), providing a potential diagnostic tool for identifying aging semen. The combination of metabolomics and proteomics analyses revealed the enrichment of energy metabolism and oxidative stress-related pathways. These findings suggest that alterations in energy consumption and increased oxidative stress could be responsible for the decline in sperm motility and DNA integrity observed with aging. Overall, this study highlights the importance of understanding changes in semen metabolome and sperm proteome with aging and their implications for male reproductive health.

Currently, testosterone replacement therapy (TRT) remains an important treatment option for hypogonadism, although its clinical application has been limited due to serious adverse effects. TRT has traditionally been associated with adverse prostate events (9). However, a recent meta-analysis of 35 studies with over 7,700 participants reported that TRT does not lead to abnormal prostate growth, and may even reduce the risk of prostate cancer (Zeng et al.). Intramuscular administration appears to be more effective in reducing the risk of prostate cancer. Although high-quality clinical research are still required to confirm this observation, it suggests that clinicians should consider the route of administration when prescribing TRT to minimize the risk of adverse effects.

In addition to TRT, seeking alternative drugs for testosterone may be a safer and more effective treatment strategy for male hypogonadism. Martinez-Arguelles et al. reported that the oral administration of small molecule peptides derived from VDAC1 can act within the HPG axis to increase testosterone levels in male rats, suggesting a promising new method of testosterone supplementation. This approach may have significant implications for the treatment of male hypogonadism, particularly in cases where traditional testosterone replacement therapy is not feasible or desirable.

Given that the decline in LC function is the key mechanism of age-related male hypogonadism (5, 6), targeting LCs for treatment may provide a fundamental solution to testosterone deficiency, which is superior to symptomatic treatments such as TRT. However, the molecular mechanisms of LC aging remain largely unknown. Luo et al. reported that LCs in both natural aging mice and diet-induced obese mice exhibit senescence phenotypes. Mechanistic studies revealed that p38 MAPK is a key molecule in Leydig cell aging. Inhibiting p38 MAPK activity has been shown to reverse Leydig cell senescence and increase testosterone synthesis, highlighting the important role of this pathway in male hypogonadism. This study deepens our understanding of the molecular mechanisms underlying LC aging and provides a potential therapeutic target for age-related male hypogonadism.

In conclusion, age-related male hypogonadism is a complex and multifactorial condition that demands further investigation. Recent advances in clinical and epidemiological research, testosterone supplementation, biomarkers for hypogonadism, semen quality, and Leydig cell aging offer promising avenues of research and therapeutic targets. By continuing to explore these areas, we can better understand the mechanisms underlying male hypogonadism and develop more effective treatments for this disease, ultimately improving the quality of life for affected men.

## Author contributions

KX and JY wrote the draft. GL, HC, RG and CD revised the text. All authors contributed to the article and approved the submitted version.
